# Trade challenges at the World Trade Organization to national noncommunicable disease prevention policies: A thematic document analysis of trade and health policy space

**DOI:** 10.1371/journal.pmed.1002590

**Published:** 2018-06-26

**Authors:** Pepita Barlow, Ronald Labonte, Martin McKee, David Stuckler

**Affiliations:** 1 Department of Sociology, University of Oxford, Oxford, United Kingdom; 2 School of Epidemiology and Public Health, University of Ottawa, Ottawa, Ontario, Canada; 3 Department of Public Health and Policy, London School of Hygiene & Tropical Medicine, London, United Kingdom; 4 Department of Policy Analysis and Public Management, Italy; Centers for Disease Control and Prevention, UNITED STATES

## Abstract

**Background:**

It has long been contested that trade rules and agreements are used to dispute regulations aimed at preventing noncommunicable diseases (NCDs). Yet most analyses of trade rules and agreements focus on trade disputes, potentially overlooking how a challenge to a regulation’s consistency with trade rules may lead to ‘policy or regulatory chill’ effects whereby countries delay, alter, or repeal regulations in order to avoid the costs of a dispute. Systematic empirical analysis of this pathway to impact was previously prevented by a dearth of systematically coded data.

**Methods and findings:**

Here, we analyse a newly created dataset of trade challenges about food, beverage, and tobacco regulations among 122 World Trade Organization (WTO) members from January 1, 1995 to December 31, 2016. We thematically describe the scope and frequency of trade challenges, analyse economic asymmetries between countries raising and defending them, and summarise 4 cases of their possible influence. Between 1995 and 2016, 93 food, beverage, and tobacco regulations were challenged at the WTO. ‘Unnecessary’ trade costs were the focus of 16.4% of the challenges. Only one (1.1%) challenge remained unresolved and escalated to a trade dispute. Thirty-nine (41.9%) challenges focussed on labelling regulations, and 18 (19.4%) focussed on quality standards and restrictions on certain products like processed meats and cigarette flavourings. High-income countries raised 77.4% (*n* = 72) of all challenges raised against low- and lower-middle–income countries. We further identified 4 cases in Indonesia, Chile, Colombia, and Saudi Arabia in which challenges were associated with changes to food and beverage regulations. Data limitations precluded a comprehensive evaluation of policy impact and challenge validity.

**Conclusions:**

Policy makers appear to face significant pressure to design food, beverage, and tobacco regulations that other countries will deem consistent with trade rules. Trade-related influence on public health policy is likely to be understated by analyses limited to formal trade disputes.

## Abstract 2

Pepita Barlow and colleagues analyze whether challenges to food, beverage, and tobacco regulations among WTO members may influence policies meant to promote healthy practices.

## Author summary

**Why was this study done?:**

- There is a debate about whether World Trade Organization (WTO) rules are invoked to prevent governments from introducing food, beverage, and tobacco regulations aimed at preventing noncommunicable diseases (NCDs).
- Select case studies have identified how countries may delay, alter, or repeal regulations in order to avoid the costs of a dispute following such a ‘trade challenge’.
- Yet little is known about how often this might occur, about which policies, and about whether there is any evidence of policy impact.

**What did the researchers do and find?:**

- We created and analysed a new dataset of trade challenges to food, beverage, and tobacco regulations among 122 WTO members during the period from 1995 to 2016.
- We found that, between 1995 and 2016, 93 food, beverage, and tobacco regulations were challenged at the WTO. ‘Unnecessary’ trade costs were the focus of 16.4% of challenges.
- We also identified 4 cases in which challenges were associated with policy change.

**What do these findings mean?:**

- Policy makers appear to face significant pressure to design food, beverage, and tobacco regulations that other countries will deem consistent with trade rules.
- However, more research is necessary to evaluate the validity and influence of challenges.

## Introduction

Noncommunicable diseases (NCDs) are now the leading cause of death worldwide, a major impediment to poverty reduction, and estimated to cost the global economy US$47 trillion over the next two decades [1]. At the highest political level, there is now recognition of the urgency of addressing NCDs: the UN Sustainable Development Goals, adopted by 193 countries in September 2015, included a target to reduce mortality from NCDs by one-third by 2030. This follows from the UN Political Declaration on the Prevention and Control of NCDs, agreed on in September 2011 [2,3]. To achieve these goals, WHO recommends ‘best buys’ for combatting NCDs, which are low-cost and potentially even revenue-generating. Many recommended policies are fiscal or regulatory, including labelling regulations on processed food and alcoholic beverages [4,5], taxes on sugar-sweetened beverages [5], and plain packaging of tobacco [6]. While there is strong evidence to support them, implementation is often challenged by vested economic interests [7].

One longstanding debate is whether trade rules and agreements are used to undermine food, beverage, and tobacco regulations aimed at preventing NCDs. This broad debate centres on binding and enforceable treaties that include rules designed to reduce barriers to cross-border trade in food, beverage, and tobacco products [8]. This pertains to a wide range of treaties including (i) the World Trade Organization (WTO) and its suite of trade agreements, (ii) regional or free trade agreements (FTAs) between at least two countries—which arose in the wake of WTO-stalemated negotiations, import much of the WTO treaties and then add further liberalisation components (so-called ‘WTO+’ measures), and often include investor–state dispute settlement chapters—and (iii) bilateral treaties applying to trade and/or investment, including bilateral investment treaties.

For example, WTO members are obliged to design new regulations and policies that are consistent with the rules set out in the Technical Barriers to Trade (TBT) Agreement (Box 1). Critics argue that invoking these rules could restrict ‘policy space’, or the freedom, scope, and mechanisms that governments have to design, choose, and implement public policies in order to fulfil health-related priorities and aims’ [9,10]. This may happen because the TBT Agreement enables foreign governments to dispute policies enacted by another government that they think violate the TBT Agreement. Formal dispute settlement procedures may mandate a change, delay, or even reversal of the policy, or compensatory trade penalties [11].

**Box 1. The WTO TBT agreement and associated proceedings**

**The TBT Agreement.** The WTO is an intergovernmental organisation that coordinates the rules of trade between its members. It comprised 164 countries in July 2017, with 21 countries awaiting accession [52]. Countries that join the WTO sign a series of accession agreements, including the TBT Agreement. The TBT Agreement is binding on all members of the WTO. The rules in the Agreement state that new measures must not introduce ‘unnecessary trade costs’ or barriers to trade, especially if the stated objective of the measure—such as protecting public health—could be achieved with a less costly alternative. In addition, governments must ensure that measures do not discriminate against foreign products (in favour of domestic producers) or between foreign producers (for example, by favouring one country over another). The TBT Agreement also strongly encourages the use of international standards, although the Agreement recognises that some international standards may not be appropriate in certain countries and regions or in new policy domains. Finally, members must meet certain transparency obligations, described as a ‘cornerstone’ (WTO, p. 24) of the TBT Agreement.

**The TBT committee and trade challenges.** Governments are obliged to notify other members of any new proposed regulation and provide sufficient information before it enters into force so that trading partners have the opportunity to receive feedback from industry and to provide comments at the TBT Committee that can be taken into account.

These notifications are then discussed at the WTO, where other country members have the opportunity to challenge policies that they think violate the TBT Agreement at bi-annual meetings of the TBT Committee in Geneva [53]. This constitutes a ‘trade challenge’.

There is, however, ongoing debate about whether rules such as those set out in the WTO TBT Agreement actually do impede NCD prevention. The TBT Agreement recognises a government’s right to pursue a range of ‘legitimate’ policy objectives, including the protection of human health, even if the regulation increases trade costs [12–14]. Only a few disputes about NCDs have arisen to date, and there is little evidence that they actually lead to policy changes [15]. Most studies were in legal scholarship and mainly regard the practical application of laws. For example, Mitchell and Voon (2011) evaluated whether WTO trade rules cohere with WHO’s recommended policies for reducing NCDs. They conclude that it is feasible to design public health policies that minimise adverse trade effects [15]. Bloche (2002) and Gostin (2008), in a study of WTO disputes, report that judges tend to defer to national authority when deciding outcomes, partly to avoid criticisms of judicial overreach [12,13], although the same is not the case with the independent tribunals overseeing investor–state disputes, which are widely regarded as procedurally flawed.

One major limitation of previous empirical scholarship is that it mainly focuses on formally adjudicated trade disputes. However, political economy analyses of conflict have observed that an emphasis on overt and formal conflicts, like formal WTO disputes, may obscure a substantially more significant ‘hidden hand’ of influence: how a challenge to a regulation’s consistency with trade rules raised in an intergovernmental committee may lead to ‘policy or regulatory chill’ effects, whereupon a government delays, alters, or abandons its policy in order to avoid the costs associated with a dispute [9,16,17]. For example, as several commentators have noted [11,18–20], governments may raise trade challenges at the WTO TBT Committee that lead to a change in policy. This can occur regardless of whether WTO rules do actually provide sufficient flexibility for introducing regulations to protect public health because governments may make claims that are invalid but are not formally scrutinised or may be uncertain in the absence of a formally adjudicated trade dispute [21].

Governments have incentives to avoid trade disputes because of the legal, administrative, and economic costs associated with defending their policies. These costs can be substantial; it was reported that Australia, for example, spent over US$50 million defending plain tobacco packaging against disputes citing WTO rules and other trade and investment treaties [22]. Philip Morris was subsequently ordered to pay the government’s legal fees (the exact amount was not revealed), but this was an unknown outcome when the Australian government had to incur its defensive costs.

Trade disputes can also carry significant political costs. Although the rules and procedures for resolving WTO disputes are designed to be non-contentious [23], governments may fear their political consequences and their impact on future trade negotiations [24]. The uncertainty and costs associated with trade disputes may especially deter developing countries, which are politically and economically dependent on trade with wealthier nations and have fewer economic resources to devote to legal and administrative costs [25,26].

Thus, analyses of trade disputes may give an incomplete account of the mechanisms and number of cases in which governments have invoked WTO rules in a trade challenge and subsequently influenced policy space for NCD prevention, especially in developing countries. Previous studies of these trade challenges comprised theoretical commentaries, case studies, and reviews. One highly cited example of a deterrent effect comes from Thailand’s proposed introduction of a traffic light–labelling system in 2006. Under pressure from the United States, which argued that the policy contravened WTO rules, the Thai government abandoned its preferred approach and implemented monochrome daily guideline labels, thereby preventing escalation to a trade dispute [19]. Relatedly, Thow et al. (2017) analysed five trade challenges to new food-labelling regulations proposed by Indonesia, Peru, Ecuador, Thailand, and Chile during WTO meetings [27]. Lencucha et al. (2016) similarly analysed arguments against plain tobacco legislation during trade disputes and predispute trade challenges at the WTO [28].

To our knowledge, the number and scope of trade challenges has yet to be systematically investigated, in part because—while textual data exist—they have yet to be codified into a quantitative dataset. This precludes a systematic understanding of the scope and frequency with which trade rules are invoked to challenge and potentially influence food, beverage, and tobacco regulations in practice [10,11,29]. Many fundamental questions therefore remain poorly understood: how common are trade challenges to food, beverage, or tobacco regulations? What proportion of trade challenges escalate to trade disputes? What products and policies are under scrutiny, what trade rules are cited, and to what degree do these challenges reflect the well-known asymmetry of economic and political power and capacity between high- and low-income countries? Is there evidence that trade challenges impact policy?

Here, we address these limitations by taking advantage of a new dataset we created comprising trade challenges to food, beverage, and tobacco regulations among 122 countries at the WTO TBT during the period from 1995 to 2016. This includes those that occurred both with and without a subsequent dispute. We thematically analyse the scope, frequency, and content of trade challenges; identify asymmetries in the economic resources and power of the countries raising and defending them; and summarise 4 case studies—selected from 4 countries that faced pressure from developed countries—that illustrate how trade challenges may be invoked in the context of the TBT framework to influence regulations targeting 3 products that are central to NCD prevention: processed foods, soft drinks, and alcohol.

## Methods

### Constructing a database of trade challenges at the WTO TBT committee

Drawing on published notifications at the WTO TBT and minutes of meetings, we constructed a dataset (DOI: 10.7910/DVN/EE5UPS) of trade challenges at the TBT Committee by combining data from 2 sources: the WTO TBT Information Management System (TBT-IMS) database and written minutes of TBT Committee meetings [30,31]. The TBT-IMS is a freely available online database of all regulations that the WTO was notified about since its foundation in 1995. The database includes information about the regulation’s original objective(s). These are coded by the country officials who registered the details of the regulation with the WTO into one of the WTO’s predefined legitimate policy goals, including ‘the protection of human health or safety’. The TBT-IMS database summarises information about the challenges, providing a unique ID for each regulation, its objective, the names of the countries that challenged that regulation, the trade rules they argued were being contravened, and dates of the TBT Committee meetings in which these challenges were raised.

To code the dataset, we extracted data about challenges raised about regulations specifically registered with the objective of ‘protecting human health or safety’. Because the TBT-IMS did not provide full details of the specific issues cited in each challenge—the type of regulation being introduced, whether it had been implemented or not, and the products it applied to—we manually extracted and coded this information from the minutes of TBT meetings in which each challenge was raised. We obtained these minutes from the WTO’s freely accessible ‘Documents Online’ repository [32]. To identify discussions about each regulation and associated challenges, we first identified and downloaded the minutes from each meeting in which the challenges were raised, as listed in the TBT-IMS database. We then identified challenges about each specific regulation using the subheadings in the minutes, which were separated according to the name and TBT-IMS ID of each regulation under discussion. From the summaries of each challenge in the relevant subsection of the minutes, we then coded the issues, type of regulation being introduced, whether it had been implemented, and the products it applied to.

To give a specific example, in May 2011, the US challenged Thailand’s new requirements for including health-warning labels on alcoholic beverage packages. The minutes from the TBT Committee meeting at which the challenge was raised stated, under the relevant subheading of the minutes, that

‘The representative of the United States raised previously-aired concerns, including the scientific basis for the text of the alcohol warning requirements, the size of the warning label in proportion to the bottle…’ (G/TBT/M/53).

Here, we coded the product as ‘beverages’ with the subcategory ‘alcohol’ and we coded the policy as ‘labelling regulations’. We identified the list of codes following widely applied procedures recommended in Miles, Huberman, and Saldana whereupon a researcher identifies a ‘start list’ of provisional codes from an initial sample of the data, takes another sample of the data, and modifies the categories after coding the second sample if necessary [33]. Further details of this coding procedure, with additional examples, are provided in S1 Text. After coding the products and other additional information, we then restricted the analytical sample to challenges that were raised about food, beverage, and tobacco products only. Finally, to study possible economic asymmetries in trade challenges, we imported country income-level data from the World Bank World Development Indicators, 2017 edition, and grouped countries into income quartiles according to their income for the year in which the challenge was raised [34]. The final analytic sample includes 93 challenges raised in the period from 1995—when the first challenge to a food, beverage, or tobacco regulation was raised—to 2016, when complete data were last available.

### Analysis

To summarise the frequency and content of trade challenges, we calculated the total number of challenges raised each year by each country per year they had been a WTO member and by countries according to income quartile. It was not possible to identify the outcome of every challenge because this was not systematically reported in the TBT-IMS or TBT Committee meeting minutes, although we could observe whether or not it escalated to a trade dispute and the number of meetings in which challenges were made towards a policy. We also were able to code the stage of a policy’s development from information contained in the meeting minutes into 3 categories: proposed but not ratified or implemented, ratified but not implemented, and in force. To study potential power asymmetries between wealthier and poorer nations, we used social network analysis to summarise the frequency of trade challenges between countries and patterns in the income quartiles of countries forming these dyads.

Finally, to assess the potential impact of challenges on policy, we evaluated, in-depth, 4 case studies in which trade challenges were associated with changes or delays to food, beverage, or tobacco regulations. These were selected based on 4 criteria. First, to assess pressure, we focused on trade challenges involving possible power asymmetries (i.e., from high-income towards lower-income countries). Second, following previous studies of international political economy, we selected those cases with heightened importance in scholarly debates [35,36]. In this literature, it involves challenges around processed foods, sugar-sweetened soft drinks, alcoholic beverages, and tobacco. Third, we assessed whether data were available to track policy changes before and after TBT meetings in the TBT minutes. Finally, we excluded the Thai traffic light–labelling case and plain tobacco packaging debates because they have already been described in extensive detail elsewhere [24,28,37]. This yielded 4 cases in Indonesia (processed foods), Chile (processed foods), Colombia (alcohol), and Saudi Arabia (soft drinks).

## Results

### Trends in trade challenges to food, beverage, and tobacco regulations, 1995–2016

First, we describe the results from our analysis of trends in trade challenges and the characteristics of countries raising and defending them. Our analysis of trade challenges at the WTO between 1995 and 2016 demonstrates that a growing number of food, beverage, and tobacco regulations are extensively scrutinised and challenged on the basis of their purported violations of trade rules. Between 1995 and 2016, a total of 93 trade challenges were raised concerning regulations aimed at protecting individuals from the risks associated with food, beverage, and tobacco products (see S1 Table). These challenges constituted 17.9% of all challenges raised at the TBT committee during this period and 38.0% of those pertaining to public health.

**Fig 1:**
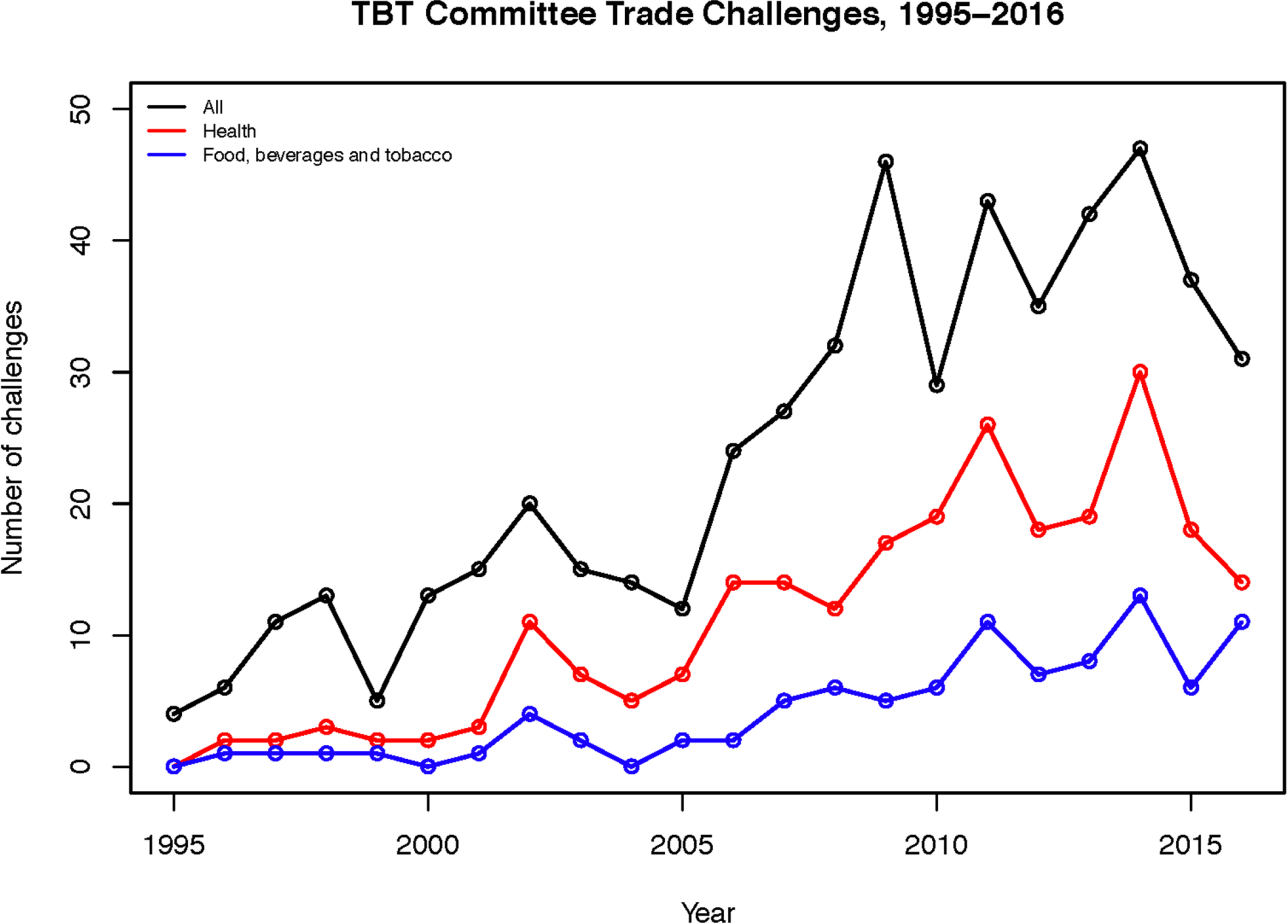
Trends in trade challenges to food, beverage, and tobacco regulations, 1995–2015. TBT, Technical Barriers to Trade.

As shown in Fig 1, the number of challenges to food, beverage, and tobacco regulations per year has grown markedly over time, rising from 0 in 1995 to a high of 13 in 2014. Few of these escalated further, suggesting that they were resolved without resorting to formal WTO channels. Escalating a challenge to a trade dispute occurred in only 1 instance (1.1% of trade challenges)—the Australian plain packaging case. Six other disputes have occurred independently of TBT challenges, citing other agreements. They ruled against, for example, discriminatory levels of taxation on foreign tobacco products (in the case of Thailand versus US under the General Agreement on Tariffs and Trade [GATT], the WTO’s precursor) and alcohol (in the case of Chile versus European Communities, under the WTO).

A majority of challenges were raised before the policy had been finalised or implemented, potentially delaying or impeding the policy altogether: 57 (61.3%) food, beverage, and tobacco regulations had been proposed or drafted but not yet ratified or implemented when the trade challenges were raised; 12 (12.9%) had been ratified but not implemented; and 24 (25.8%) were already in force. In addition, a majority of challenges were raised more than once: they were raised during 1 meeting in 29 (31.2%) cases, during 2 meetings in 20 (21.5%) cases, and during at least 3 meetings in 44 (47.3%) cases.

### Member characteristics and power asymmetries

The European Union (EU), the US, Mexico, and Canada most frequently raised trade challenges about food, beverage, and tobacco regulations. The highest-income members raised just under half of all challenges (42 [45.2%] challenges), followed by upper-middle–income members (26 [27.9%] challenges), lower-middle–income members (15 [16.1%] challenges), and low-income members (10 [10.8%] challenges). Trade challenges were most frequently raised against the EU (*n* = 11), Brazil (*n =* 9), Ecuador (*n =* 6), and Canada (*n =* 3). Overall, just under half were raised against the highest-income members (41 [44.1%] challenges), followed by lower-middle–income members (15 [16.1%] challenges), upper-middle–income members (14 [15.1%] challenges), and low-income members (22 [23.7%] challenges). The 5 most common challenge dyads were the EU against Brazil (*n =* 8), Canada against Ecuador (*n =* 5), the EU against Ecuador (*n =* 5), the US against Brazil (*n =* 5), and the US against Ecuador (*n =* 5).

**Fig 2:**
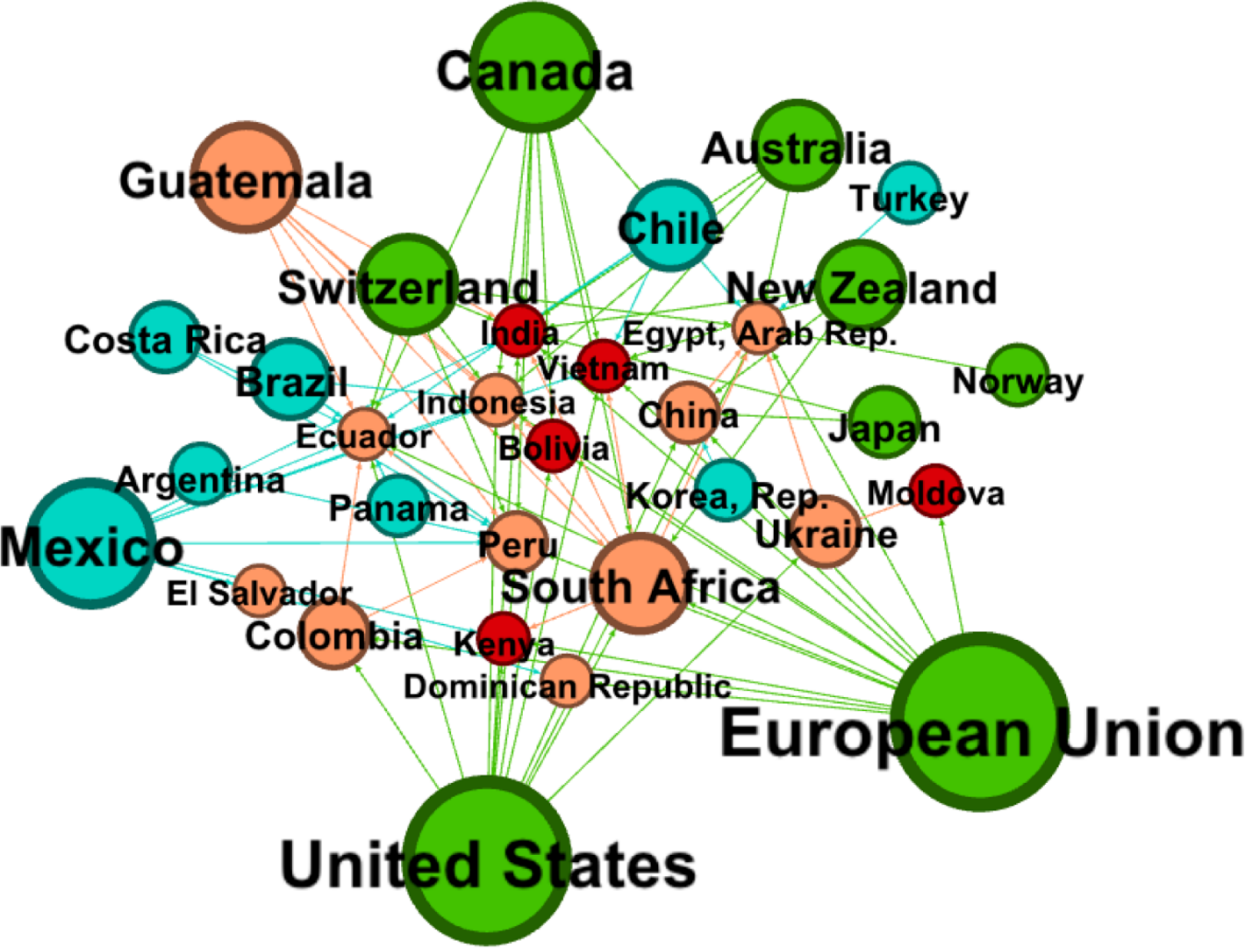
Trade challenges raised against low- and lower-middle–income countries. Notes: node colour represents country income levels, grouped into quartiles from lowest to highest. Red = Q1, orange = Q2, blue = Q3, and green = Q4. Lines show challenges raised by and to each node. Node size corresponds with the number of challenges raised (min = 1; max = 12). The proximity of nodes to one another corresponds to the frequency of challenges raised against another node (min = 1; max = 5).

While, overall, high-income members most frequently raised and defended challenges, this obscures substantial differences in countries’ incomes within each challenge dyad. There were marked economic power asymmetries: although high-income members, especially the EU and the US, most frequently raised and defended trade challenges, over three-quarters of challenges raised against low- and lower-middle–income countries were raised by high-income countries. Thus, high-income members received challenges from other high-income members (19 [20.4%] challenges), from upper-middle–income members (28 [30.1%] challenges), from lower-middle–income members (19 [20.4%] challenges), and from low-income members (27 [29.0%] challenges) in roughly similar proportions. However, as shown in Fig 2, a large majority of challenges (*n =* 72, or 77.4%) made to low- and lower-middle–income members were raised by high-income members, especially the EU, the US, and Canada, followed by upper-middle–income members (9 [10.0%] challenges) and then by other lower-middle–income members (6 [6.5%] challenges) as well as low-income members (6 [6.5%] challenges).

### Thematic analysis

Next, we describe the policies subject to trade challenges, the products involved, and the trade rules that other countries argued were violated. As shown in Table 1, the regulations targeted by trade challenges were diverse. These included infant milk formulae, alcoholic beverages, soft drinks, manufactured food products and their ingredients, cigarettes, tobacco, and cigarette flavourings.

**Table 1:** Food, tobacco, and beverage products that were regulated by measures later subject to trade challenges.

**Product**	**Description**	**No. challenges**
**Food**	Food products, including processed foods and their ingredients	46
**Beverages**	Alcoholic beverages, soft drinks, fruit juices and other nonalcoholic beverages, infant milk formulae	36
**Tobacco**	Tobacco, tobacco flavourings, cigarettes	15

Notes: number of trade challenges about each product category exceeds the number of trade challenges (*n =* 93) because some challenges were raised about regulations that affected more than one product.

**Fig 3:**
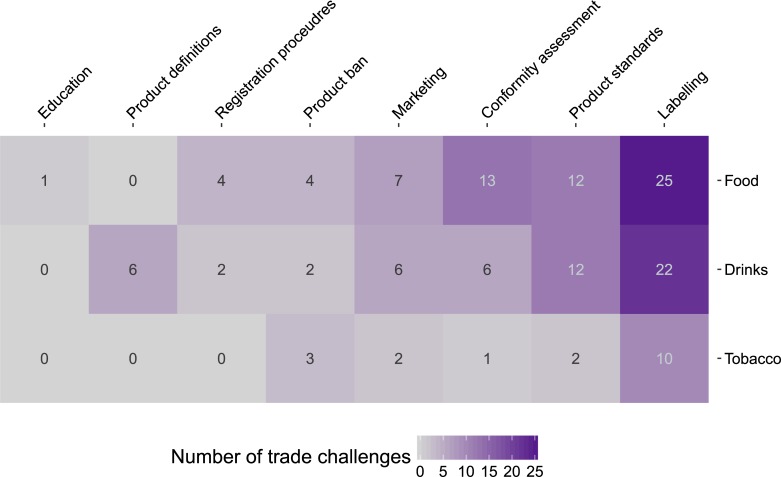
Food, beverage, and tobacco measures subject to trade challenges. Notes: see S2 and S3 Tables for full description of policies.

As shown in Fig 3, the regulations most frequently challenged were labelling requirements (39 [41.9%] challenges), product quality standards and restrictions on the use of certain ingredients (18 [19.4%] challenges), ‘conformity assessment procedures’ used for determining whether an import conformed with a given country’s regulations (12 [12.9%] challenges), and marketing regulations (10 [10.8%] challenges).

There were diverse arguments raised to challenge the health policies. The most frequent argument was that the regulations posed an ‘unnecessary barrier to trade’ (15 [16.1%] challenges), meaning that they thought the goal of the regulation could be achieved through an alternative policy or regulation that would pose fewer restrictions on trade. For example, in 2013, Mexico challenged Peruvian legislation that mandated health warnings on a range of food and beverage products high in salt, sugar, and fat and prohibited their sale in schools. Mexico argued that the legislation created ‘unnecessary’ trade costs and suggested that ‘daily meal guides’ could be developed instead (G/TBT/W/60). In 2014, the EU and the US challenged Thailand’s proposal to introduce graphic health-warning labels on alcoholic beverage packages by asking Thailand to ‘take into consideration less trade restrictive measures or, failing this, clarify on which basis and evidence Thailand concluded that different, less costly and burdensome alternatives than the indication of a graphic health warning would be insufficient to address the objective pursued’ (G/TBT/N/THA/437, G/TBT/W/64). WTO members also frequently sought clarification about an aspect of the regulation, such as the scope of products affected or the timeline to implementation (14 [15.1%] challenges).

The third most common category actually did not cite a specific violation of WTO rules (12 [12.9%] challenges), unlike the other challenges. Alternatively, it focused on how the health policy posed a barrier or cost to trade—although the argument did not state that this was ‘unnecessary’ (5 [5.4%] challenges). Another common argument was when WTO members claimed that there were other harmful and unintended consequences, such as causing consumer confusion or increasing unemployment (4 [4.3%] challenges), or questioned whether the costs of the regulation were proportionate with the risks associated with the products (4 [4.3%] challenges). In other cases, WTO members argued that the scientific evidence supporting the measures were inadequate (4 [4.3%] challenges). This complaint was raised against a range of regulations, including tobacco packaging requirements, alcoholic beverage labelling regulations, marketing and labelling requirements for energy drinks, and regulations for front-of-pack nutrition labelling for diverse food products.

Fourth, WTO members often sought greater transparency in another country’s reporting procedures (11 [11.8%] challenges), meaning that the country had provided no or little information about a measure or had left inadequate time for other countries to adapt to the new regulations. In addition, members frequently questioned the regulation’s ‘rationale or legitimacy’, or why the measure was being introduced in the first place (11 [11.8%] challenges).

Fifth, WTO members regularly challenged regulations by arguing that they were inconsistent with existing international standards, as required by WTO rules (10 [10.8%] challenges). For example, on at least one occasion, members challenged regulations that were based on WHO guidelines because these are not officially classified as international standards. For example, in 2016, the US challenged Thailand’s proposal to introduce mandatory restrictions on the content and marketing of a milk formula for infants. A Thai official noted that the measure was based on WHO’s International Code of Marketing of Breast-milk Substitutes and a 2016 World Health Assembly resolution on ending the inappropriate promotion of foods for infants and young children. The US raised its objections about the costs of the measure by arguing that the WHO guidance ‘was not an international standard in accordance with the criteria established by the TBT Committee’ and that ‘the application of the 1981 WHO's International Code of Marketing of Breast-milk Substitutes was voluntary’ (G/TBT/W/69).

### Case studies of influence on policy space for the prevention of NCDs

Here, we summarise case studies of a possible influence of trade challenges on policy. We identified 4 country case studies from Indonesia, Chile, Colombia, and Saudi Arabia that met our selection criteria as trade challenges from high-income members that were associated with changes or delays in food and beverage regulations. First, in 2013, the Indonesian government ratified legislation that mandated the display of health warnings on processed food packages from April 2016 onwards (G/TBT/N/IDN/84). The warnings would describe the product’s salt, sugar, and fat content. Several members, including Canada, Australia, and the EU, challenged the measure at the WTO TBT Committee, questioning the scientific justification for the measure and requesting that Indonesia consider an alternative measure that would be less trade burdensome. Later, Indonesia reported to the TBT Committee that the regulation’s scheduled implementation had been delayed by 4 years to 2019 (G/TBT/W/67). Indonesia also said that it would reevaluate the regulation and consider alternative approaches to NCD prevention during the extended transition period. This included considering a public education campaign to increase awareness about the risks associated with excess sugar, salt, and fat consumption instead of imposing mandatory labels, as a representative of the EU had suggested at an earlier WTO meeting (G/TBT/W/60).

Second, in 2013, Chile proposed legislation that would mandate the display of a ‘STOP’ sign on the packages of food and beverage products high in salt, sugar, and/or fat. The US, Australia, Switzerland, Canada, Argentina, and the EU, among others, objected to the measure on 8 occasions between 2013 and 2016. They challenged the basis for deciding the size and colour of the sign, which was to be red or black and occupy at least 20% of the main face of the packaging (G/TBT/W/59). Chile later reported to the TBT Committee that it had made substantial changes to the labelling requirements, reducing the required size of the warning label, and had considered allowing producers to choose between a wider range of colours—including green (G/TBT/W/62), a colour promoted for voluntary use by a food industry consortium as indicative of a healthy food choice [38]. Chile also announced that the measure’s scheduled implementation was to be delayed by 3 years from 2013 to 2016. The final measure retained the black-only stop sign but reduced it in size to 4%–7% of the package surface [39]. Companies have 3 years in which to comply with the Chilean legislation, which is now considered the most extensive food labelling legislation in the world. The domestic and international food industry is strongly opposed to it, fearing it will diffuse across the Latin American region, and there remain concerns that the Chilean legislation may trigger a dispute under TBT or intellectual property rights rules at the WTO or an investor–state dispute under investment treaties.

Third, in 2009, Colombia drafted a regulation that would require the display of health warnings about the risks associated with excess alcohol consumption, especially among pregnant women, on the front label of alcohol packages. In June 2009, the EU and the US complained that the warning labels would have a burdensome and costly impact on its exports to Colombia (G/TBT/W/53). The two countries raised this challenge at 4 additional TBT meetings and, in 2013, Colombia notified the WTO that the measure had been substantially amended. The final regulation significantly reduced the scope of alcohols it affected and no longer imposed any health-warning labelling requirements (G/TBT/W/55).

Finally, in 2014, the Saudi Arabian government ratified a regulation that mandated the display of health warnings on energy drink cans. Several members, including the EU, US, and Switzerland, questioned whether the health warnings were necessary and scientifically accurate (G/TBT/W/64). In 2017, the Saudi Arabian government implemented the labelling law, which required a warning stating that ‘energy drinks [are] harmful to health’ [40], but only after an official representing Saudi Arabia announced that the scheduled implementation of the measure had been ‘delayed more than once in response to the concerns of WTO Members’ (G/TBT/W/66).

## Discussion

Our systematic analysis of trade challenges at the WTO between 1995 and 2016 demonstrates that a growing number of food, beverage, and tobacco regulations are extensively scrutinised and challenged on the basis of their purported violations of trade rules. This yielded at least 4 important findings. First, the regulations targeted by trade challenges were diverse, including those dealing with infant milk formulae, alcoholic beverages, soft drinks, manufactured food products and their ingredients, cigarettes, tobacco, and cigarette flavourings. Second, countries most frequently challenged one another’s labelling regulations and product quality standards or restrictions on using certain products such as processed meats or cigarette flavourings. Members often challenged the additional costs of the measure, requested more information, and sought greater transparency in other country’s notification procedures. Third, there were marked power asymmetries: although high-income members—especially the EU and the US—most frequently raised and defended trade challenges, over three-quarters of challenges raised against low- and lower-middle–income members were raised by high-income members. Finally, trade challenges were often raised on multiple occasions before the regulation was ratified or implemented, potentially delaying or impeding the policy altogether. In 4 country case studies from Indonesia, Chile, Colombia, and Saudi Arabia, trade challenges were associated with changes or delays in food and beverage regulations. In the case of Chile and Saudi Arabia, these countries did eventually proceed with new laws. It remains possible, however, that both countries—and especially Chile—may face a formal WTO dispute over their policies.

It has long been debated whether governments invoke trade rules to limit policy space for preventing NCDs, leading to ‘policy or regulatory chill’ effects, whereupon a government delays, alters, or abandons its policy in order to avoid the costs associated with a trade dispute [10]. To our knowledge, our study is the first to systematically analyse this potential pathway to influencing public health policy. Our study has demonstrated that countries regularly face policy pressures at the WTO TBT Committee; that compliance with complaints is frequently monitored; and that this pressure may have been influential in delaying, altering, or abandoning food, beverage, and tobacco regulations. These results do not imply that WTO rules provide insufficient flexibility for regulations to promote various policy interests, including public health. Instead, our analysis shows that governments regularly invoke trade rules to pressure other countries to change their food, beverage, and tobacco regulations.

Our study also builds on prior research by revealing significant power asymmetries: a majority (*n =* 72, or 77.4%) of challenges raised against low- and lower-middle–income countries was raised by wealthier nations. The dominant players were the US, China, and the EU, countries often seen as competing for global, political, and economic leadership [41]. At the WTO, country representatives also stated occasionally that their challenges represented the interests of the food and beverage industry. For example, in 2013, a representative from the US commented on Peru’s attempts at introducing health warnings on select food and beverage products by stating that ‘the US pre-packaged food industry has expressed concern over the economic impact of the inclusion of warning statements on a mandatory basis’ (G/TBT/W/60). Similarly, in 2006, a Canadian representative commented on Thailand’s proposed snack food labelling regulations by noting that ‘the Canadian industry had questioned the scientific merit of the proposed regulation and argued that it discriminated against snack foods’ in a letter to the Canadian government (G/TBT/W/43). Thus, like other international fora [42–44], the TBT Committee may serve as a forum in which power asymmetries are exerted and translate into policy leverage. However, it is important to note that WTO rules may also provide lower-income countries with an opportunity to raise challenges against wealthier countries that might have otherwise been unavailable [45]. This could explain why one-third of challenges against high-income countries were raised by low-income countries.

Our approach also demonstrates the importance of evaluating potential regulatory or policy chill that occurs outside trade disputes and may occur at earlier stages of policy making in intergovernmental trade committees. Only 1 (1.1%) of the 93 challenges to food, beverage, and tobacco regulations at the WTO later escalated to a formally adjudicated trade dispute—the Australian plain packaging case. Insofar as challenges impact policy, this suggests that the influence of trade rules on NCD prevention via regulatory or policy chill effects is much more extensive than that indicated by the outcomes of trade dispute rulings.

Our study has several limitations, arising from the nature of the data, which we have sought to address. It was not possible to identify whether and how all 93 trade challenges ultimately impacted policy because this information is not systematically reported in the TBT-IMS or the TBT Committee minutes. Our analysis nevertheless suggests that at least some trade challenges may have been influential. Most regulations had not yet been ratified or implemented when challenges were raised, so a change in policy was actually possible. A majority of trade challenges were also raised repeatedly. This suggests that those raising trade challenges subsequently monitored adherence to their complaints and expected that their concerns would be addressed. In addition, repeated challenges may have increased the incentives for complying with complaints if they were interpreted as a signal that a politically and economically costly trade dispute was increasingly likely. Furthermore, we identified 4 cases in which trade challenges were associated with a reported delay or change in policy. Whilst we cannot definitively confirm that informal WTO challenges led to these policy changes, the sequencing of these events—and the incorporation of comments made at the WTO into the final policy—are indications of influence.

Fourth, we did not evaluate or grade the evidence base associated with public health policies. However, many of them were WHO-recommended policies. We also did not investigate the validity of trade challenges citing trade rules. Trade challenges may constitute a legitimate complaint about a policy according to TBT rules because, for example, the regulations were unclear or created additional costs without improving effectiveness. In addition, the applicability of trade rules and associated validity of trade challenges may be uncertain due to, for example, how scientific ambiguity regarding the effectiveness of regulations and their optimal design is understood in relation to the TBT Agreement or due to the absence of clear international standards, norms, and guidelines. The challenges may also be invalid because they cite TBT rules where they did not apply. This could include, for example, making false scientific claims that a proposed policy is ineffective (e.g., plain tobacco packaging) and/or claiming that an alternative is more effective (e.g., daily meal guides and educational campaigns are a more effective means to reduce obesity than the proposed regulation). Alternatively, the challenges may be valid, and the rules genuinely restrictive, when governments seek to introduce regulations without a strong scientific basis and minimal trade cost.

Fifth, it is still possible that regulatory chill occurs without a visible challenge at the TBT committee due to, for example, the threat of a dispute made outside the WTO, which is not revealed in WTO documents [19]. Finally, our study may have limited external validity beyond the TBT Committee and WTO dispute process. This includes disputes within the EU, in which the European Court of Justice has consistently applied the requirement in the European Treaties to ensure a high level of health in all policies [46]. In addition, our study may not represent the diverse scope and content of challenges raised under alternative trade and/or investment agreements, such as bilateral and regional treaties and other agreements that grant legal protections to investments as well as liberalise trade in goods and services and that extend those rights to foreign investors (including multinational corporations) as well as to governments [19]. Nonetheless, our results may be informative elsewhere because government officials frequently stated that trade challenges at the WTO represented concerns raised by food, beverage, and tobacco corporations, many of whom make substantial foreign investments [8]. In addition, WTO debates may provide an insight into the challenges that may be raised elsewhere because the clauses in many subsequent agreements built upon and expanded WTO rules [14,47,48].

Future research should address these limitations by evaluating whether challenges are invoked citing other trade agreements and outside trade committees, the validity of trade challenges and their empirical bases, whether challenges undermine effective public health policies, and whether challenges raised by low- and lower-middle–income countries were as effective as those raised by upper-middle–income and high-income countries. In addition, more research is necessary to investigate the determinants of challenge dyads. For example, a country may raise a WTO dispute due to high levels and projected growth rates of bilateral trade and investment, increasing a country’s interest in the introduction of a new policy and its potential effects. Alternatively, challenges may not correlate with trade flows and instead represent an effort to ensure that trade costs on all products are kept to a minimum and so prevent the introduction of costs on the specific products that a country trades in. More research is also needed to investigate why specific countries are frequently subject to trade challenges. Ecuador, for example, was featured 6 times in TBT Committee challenges despite its small size, potentially owing to persistent violations of TBT rules or its high levels of activity in implementing food, beverage, and tobacco regulations to prevent NCDs [54].

Our study has important implications for NCD prevention. There is now substantial evidence both that mandatory measures addressing price, availability, and marketing of food, beverage, and tobacco products are the most effective low-cost tools for preventing and controlling NCDs and that these measures are most aggressively opposed by the global corporations that manufacture them [49]. Thus, governments—informed by the increasing international exchange of ideas—are seeking to introduce them at a time when new trade deals are being negotiated at an unprecedented rate. Whereas tariffs—a type of trade tax—were previously the mainstay of trade negotiations, product rules and regulations are now regarded as constituting the greatest barriers to international trade in many industries [50]. For EU–US trade as a whole, for example, regulatory differences are estimated to have a trade-dampening effect equivalent to a 30% import tax [51]. Harmonising regulatory differences, including those in the food, beverage, and tobacco sectors, is therefore a central goal of contemporary trade policy [48]. Our study shows that countries have regularly argued that one another’s food, beverage, and tobacco regulations—including those targeting NCDs—violate trade rules despite the presence of health exceptions in WTO rules, suggesting that these may be renegotiated under new, as well as existing, trade agreements.

Finally, our results have implications for trade policy and negotiations. The fact that WTO rules are regularly used to challenge new food, beverage, and tobacco regulations suggests that partners in other trade agreements may invoke their rules to challenge one another’s policies targeting NCD reduction. A related body of research on the effects of trade rules and agreements found that they can contribute to substantial rises in the availability, affordability, and promotion of tobacco, food, and beverages that increase the risk of NCDs [18,29]. This suggests that new trade agreements could undermine NCD prevention by encouraging higher consumption of harmful food, beverages, and tobacco products whilst also being invoked to hinder a government’s ability to introduce new legislation aimed at reducing consumption. Trade negotiations may be a critical window for ensuring that trade rules help rather than hinder policy makers’ commitments to prevent and control NCDs.

## Conclusions

Our systematic analysis of trade challenges at the WTO between 1995 and 2016 demonstrates that a growing number of food, beverage, and tobacco regulations are extensively scrutinised and challenged on the basis of their purported violations of trade rules. This pressure may have been influential in delaying, altering, or abandoning food, beverage, and tobacco regulations. Our study also revealed significant power asymmetries: a majority (77.4%) of challenges raised against low- and lower-middle–income countries was raised by high-income countries. These findings show that policy makers appear to face significant pressure to design food, beverage, and tobacco regulations that other countries will deem consistent with WTO rules and that policy making in low- and lower-middle–income countries may face pressure from the economic and political interests of wealthier nations.

## Supporting information

S1 Text Constructing a novel database of informal WTO challenges: Data sources and codebook. (DOCX)

S1 Table Summary of trade challenges to food, beverage, and tobacco regulations. (DOCX)

S2 Table Measures introduced by countries to protect health that were subject to trade challenges. (DOCX)

S3 Table Issues raised in informal challenges about measures introduced to protect health. (DOCX)

## Abbreviations

EU: European Union
FTA: free trade agreement
GATT: General Agreement on Tariffs and Trade
NCD: noncommunicable disease
TBT: Technical Barriers to Trade
TBT-IMS: Technical Barriers to Trade Information Management System
WTO: World Trade Organization
