# Supplementary material for: Trade challenges at the World Trade Organization to national noncommunicable disease prevention policies: A thematic document analysis of trade and health policy space
Source: PLoS Med. 2018 Jun 26;15(6):e1002590. doi: 10.1371/journal.pmed.1002590 (PMC6019096; doi:10.1371/journal.pmed.1002590)
Supplement: S1 Text — (DOCX) [file pmed.1002590.s001.docx]

**S1 Text. Constructing a novel database of informal WTO challenges: data sources and codebook**

*The TBT Information Management System*

The TBT Information Management System (‘TBT-IMS’) is an online platform developed by the WTO to facilitate the fulfilment of members obligations under the TBT Agreement of notifying the WTO about any new technical regulation or other measure that might affect trade (WTO 2017b). Members use the TBT-IMS to register a brief description of the measure and its objectives, including whether it was intended to ‘protect human health or safety’. Notified measures can then be monitored by other members. If they wish, the other members can register a ‘specific trade concern’ (STC) about the measure on the database, including the specific type of issue posed by the measure. These STCs are later discussed at tri-annual TBT Committee meetings.

The full TBT-IMS database provides a unique source of information about the details of each STC – or, what we call an ‘informal challenge’ – that was raised about regulations and other measures intended to ‘protect human health or safety’. The database details the country implementing the measure subject to the STC, its objectives, the countries raising the STCs, the dates of committee meetings where the STCs were raised, together with a unique ID (‘IMS ID’) for each notified measure.

*Policies, stage in development, and products affected*

As details of the specific policies being challenged, the products the policies affected, and the policy’s stage in development (e.g. draft or already implemented) were not universally available in the TBT-IMS we manually extracted and coded this information from the 1,771 pages of the minutes from all 69 TBT Committee meetings that took place, 1996-2016. We identified the relevant STC and associated policy from the TBT-IMS in TBT Committee meeting minutes by first identifying the dates of committee meetings where the STC was raised - as indicated in the TBT-IMS database. We then used this to identify the minutes from the TBT Committee meeting on that date meeting in the ‘Documents Online’ database, which provides the official minutes of the WTO's councils, committees, and working groups (WTO 2017a).

The minutes of TBT Committee meetings were broken down into multiple sub-headings, with one sub-heading for each policy about which STCs were raised. This sub-heading refers to the unique IMS ID of the notified measure, allowing us to link discussions about a policy in the minutes with TBT Minutes with the TBT-IMS database.

Finally, as members sometimes submitted written comments in addition to raising their concerns orally at TBT Committee meetings, we also sourced these written comments from the ‘Documents Online’ database using the unique document ID referenced in the minutes.

*Coding procedures*

When coding the data our goal was to describe succinctly the content of each policy, policy stage and product in each dispute by assigning them exhaustive categories. Specifically, each code was intended to answer a specific set of questions. These were: ‘at what stage of development was the policy when the TBT was raised?’ (policy stage), ‘what product(s) did the policy affect that the concern was raised about?’ (product), and ‘what was the specific policy about which the STC was raised?’ (policy).

To code the policies, products and policy stages we followed widely applied methods recommended in Miles and Huberman (1994, 2015). Briefly, this involves 1) identifying a ‘start list’ of provisional codes based on relevant literature, 2) coding a sample of the data whilst adding or modifying the codebook where appropriate, 3) coding the remaining data, again modifying the codebook where necessary, and 4) checking for coding accuracy and reliability. Table 1 summarises the codes as identified and assigned using the above procedure, which is outlined below in further detail.

In the first stage we identified a provisional ‘start list’ of codes for describing the policies, products and the policy’s stage of development from relevant WTO legal documents. These were, the ‘WTO Agreement Series: Technical Barriers to Trade, 2015 edition’, used for identifying policies and the different stages in a policy’s development when a dispute can arise, and section headings in the 2002 ‘Harmonised Commodity Description and Coding System (Harmonised System, or HS) Combined Nomenclature, 2016 edition’, used for identifying product categories.

In the second sage we selected a sample of 10 challenge cases and identified the meetings and the relevant minutes from which the challenges were raised. The minutes were separated into different headings referring to each challenge, followed by a summary of the comments by each country raising the challenge and a response from the country subject to the challenge. Next, we extracted information about the policy, policy stage, and product and assigned them into categories using the start list of codes. These codes were identified from the comments made by members about the challenge, as shown in the ‘Examples’ column in Table 1 below.

At this stage, we also modified and disaggregated existing codes whilst adding new codes when the pre-defined codes were not appropriate, were ambiguous, or combined a large number of diverse policies, policy stages, or products into a single category. For example, it was not possible to identify the policy stage in the level of detail required to code into the 9 distinct categories identified in our start list so we collapsed these stages into four policy development stage categories capturing 4 stages from proposal to implementation: proposed, drafted, ratified but not implemented, and implemented. In addition, some product categories were disaggregated. For example, we separated food, beverages, and tobacco into separate groups.

At the third stage we used the codes developed from the first two stages to code the remainder of the data. Again, we modified existing codes and developed new codes where necessary - revising previously coded data with the updated codes and codebook as appropriate. For example, we reduced the number of ‘policy stage’ codes once again from 4 to 3 codes as it was sometimes ambiguous whether a policy was ‘proposed’ or ‘drafted’, and WTO members sometimes referred to a policy using both words interchangeably. We therefore collapsed this into one code: ‘proposed or drafted’.

Finally, to check the reliability of the codes we randomly selected 10% (n = 25) of challenges which were then coded by another researcher at the lead author’s institution using the codebook developed from the procedures described above. Ambiguities in coding arose in n = 3 cases and were resolved via discussion by updating the codebook to expand the definitions.

*‘Other issues’ raised in food, beverage and tobacco policy challenges*

When raising concerns about a policy at TBT Committee meetings WTO members register the details of their concern on the TBT-IMS. When doing so they provide details of the specific issue or issues posed by the measure. These issues include violations of the TBT Agreement which are registered in predefined categories on the TBT-IMS. However, WTO members frequently flagged ‘other issues’ alongside their concern about possible TBT violations. As these were among the most common type of issue raised against the policies we manually coded the specific content of these ‘other issues’. As we could not identify a provisional ‘start list’ for coding this open-ended and ambiguous category of issues we coded them inductively.

First, we selected a sample of 10 challenges from the dataset and read the minutes from TBT Committee meetings about each challenge. We identified the issues raised by members at the TBT Committee meetings that did not fall into the other categories of trade rule issues that they raised. Next, we developed a provisional codebook for describing these ‘other issues’ by succinctly describing each issue in each challenge, searching for common types of issue in the sample, developing categories of codes, and then coding each issue using these categories. Finally, we used the codes developed from the first two stages to code the remainder of the data. Again, we modified existing codes and developed new codes where necessary - revising previously coded data with the updated codes and codebook as appropriate.

Table 1. Policy, policy stage, and product codes

| Variable | Code | Description | Example |
| --- | --- | --- | --- |
| Policy | Labelling | Regulations for product packaging and labelling | “The representative of the United States raised previously-aired concerns, including the scientific basis for the text of the alcohol warning requirements, the size of the warning label in proportion to the bottle…” (G/TBT/M/53)  “The representative of Argentina reiterated his delegation's concerns with respect to the EC regulation on production and labelling of organic products…” (G/TBT/M/45) |
|  | Product standards and restrictions | Product standards, including production and quality requirements and maximum limits on the use of certain materials in their production | “The representative of the European Union raised concern regarding Colombia's draft decree laying out requirements for the manufacture, processing, packaging, marketing, sale, export and import of alcoholic beverages…she noted that some quality parameters, such as the restriction on the use of colouring, flavouring and sweeteners in liqueurs were not in line with international practice…” (G/TBT/M/53) |
|  | Conformity assessment procedures | Regulations for certifying that products conform to a country’s regulations | “The representative of the United States raised concerns with respect to Israeli Ministry of Health regulations governing the sale of infant formula… He pointed out that the US TBT Enquiry Point had sent two separate requests on 10 April and 15 May 2007 for copies of all relevant Israeli Ministry of Health regulations … on infant formula laboratory testing, renewal of infant formula product licences and administrative fees…” (G/TBT/M/42) |
|  | Product ban | Full prohibition of a product applying both to importation and domestic production | “The representative of Argentina raised a concern regarding Canada's legislation "Cracking Down on Tobacco Marketing Aimed at Youth Act"… The representative of Argentina noted that the measure prohibited the use of various additives in certain tobacco products, including cigarettes, cigarillos and blunt wraps” (G/TBT/M/49)  “The representative of Australia said that in December 2014, the Indonesian Government had issued a regulation limiting imports of beef to prime cuts and some offal and manufactured meat.” (G/TBT/M/65) |
|  | Product definitions | Regulations about the definitions and/ or classification of a specific product | “The representative of the European Union stated that in January 2016 the EU had provided detailed comments on India's draft alcoholic beverages regulation (notified in December 2015) establishing the requirements and definitions applicable to different types of alcoholic beverages (such as spirits, wines and beers)…” (G/TBT/M/68)  “The representative of Barbados drew attention to a Brazilian notification circulated on 27 October 2003 concerning a decree setting out amendments to definitions for certain beverages and spirits, specifically aguardiente, cachaza and rum…” (G/TBT/M/31) |
|  | Registration | Regulations for registering a product for production, sale and/or marketing in a country | “The representative of Mexico stated that the Congress of the United States had approved the Food Safety Modernization Public Law in January 2011, and that the promulgation provided for major changes in the areas of production, transportation, distribution and import of food products into the United States…This covered the gathering of records of all involved establishments, and the registration of these establishments with the FDA when they were part of the food production chain.” (G/TBT/M/53)  “The representative of Mexico noted her delegation's concern with Brazil's draft technical regulation on the list of foods that needs to be registered before marketing.” (G/TBT/M/50) |
|  | Education | Policies to promote education through an education campaign | “The representative of Peru stated that the measure's objective was to reduce obesity and other risks to non-communicable diseases through: organizing educative campaigns in schools to promote healthy eating….” (G/TBT/M/60) |
| Policy stage | Drafted or proposed | The measure had been proposed and/or a first draft of the measure had been written but was not yet finalised, approved, and/or passed into law | “The representative of Zimbabwe said that her delegation shared the concerns raised by the previous delegations regarding Ireland's proposal to introduce standardized plain packaging of tobacco products…” (G/TBT/M/60)  “China - Formula Registration Regulation for Infant and Follow-up Formula, G/TBT/N/CHN/1165 (IMS ID 493) …. The representative of the European Union continued to have serious concerns about some aspects of the draft measure.” (G/TBT/M/69) |
|  | Ratified | The measure had been finalised, approved by government authorities, and/ or passed into law, but had not yet come into effect | “Ecuador - Resolution No. SENAE-DGN-2013-0300-RE relating to post entry control of imported alcoholic beverages… the EU expressed its concern with the entry into force of the measure, as it allowed just 30 days before beverages shipped to Ecuador would be required to comply with the labelling obligation…” (G/TBT/M/62)  “The representative of Mexico stated that the Congress of the United States had approved the Food Safety Modernization Public Law in January 2011, and that the promulgation provided for major changes in the areas of production, transportation, distribution and import of food products into the United States….” (G/TBT/M/53 |
|  | Implemented | The measure had already come into effect when the challenge was raised | “The representative of Mexico expressed concern about the Alcoholic Controls Act 2010, published by Kenya on 7 December 2010, and notified 1 March 2011… she said Kenya did not appear to uphold the obligation stemming from Article 2.9 of the TBT Agreement, since the legislation entered into force before it had been notified to the WTO: the measure was notified in March 2011 but had been enforced in December 2010.” (G/TBT/M/54)  *“*China - Specification for Import and Export of Food Additives Inspection, Quarantine and Supervision (2011 No. 52) – Disclosure of formulas for imported food additives... The representative of the United States recalled that this measure was enacted by China's General Administration of Quality Supervision, Inspection and Quarantine (AQSIQ) on 18 April 2011 and it had entered into force on 1 July 2011.” (G/TBT/M/55) |
| Product | Food | Regulations that affect processed and un-processed foods, and their ingredients, including sugar, fat, salt and genetically modified foods and ingredients | “The representative of the United States raised concerns regarding Korea's Ministerial Enforcement Regulation for Food Industry Promotion Act… The new measure, which included guidelines, regulations and certification procedures for organic processed foods…” (G/TBT/M/48)  “Indonesia – Labelling Regulations (Ministry of Trade Regulation 62/2009 and 22/2010…The representative of the United States said that the supplementary labelling for processed food products should be allowed to be applied at the importers' select warehouse and other approved locations” (G/TBT/M/55) |
|  | Beverages | Alcoholic beverages, soft-drinks, juices and infant milk formulae | “The representative of the European Communities was concerned about a measure adopted by Moldova on 15 August 2007 (Government Decision 934) which introduced new quality and control requirements for bottled, non-alcoholic beverages including mineral, natural water and soft drinks.” (G/TBT/M/43)  “The representative of the United States expressed his delegation's concerns on the proposed warning label requirements for alcoholic beverages.” (G/TBT/M/55) |
|  | Tobacco | Tobacco, tobacco additives and flavourings, and cigarettes | “The representative of the European Union reiterated concerns about Canada's Bill C-32 amending the Tobacco Act, and requested Canada to provide replies to several questions… In particular, the European Union urged Canada to provide some background with regards to its approach to ban a comprehensive list of additives, including certain flavours….” (G/TBT/M/51)  “The proposed measure would imply discontinuation of European exports of traditionally blended tobacco products to Brazil, and would also affect European exports of additives that were currently used in tobacco products.” (G/TBT/M/55) |
| ‘Other’ issues | Trade costs | Trade delays, costs or problems for businesses from implementing measure and/or its overall impact on trade | “The representative of the European Union stressed that adaptation to the new requirements would require significant investment for manufacturers…” (G/TBT/M/59)  “The representative of Canada was concerned that this regulatory proposal would have a negative impact on Canada's significant food exports to Chile…” (G/TBT/M/59) |
|  | Scientific basis | Query or concern about whether there is sufficient scientific evidence to support the introduction of the measure and achieve the stated objective of protecting human health | “The EU recognised that for certain nutrients there was evidence of a positive association between its intake and the risk of developing a disease or disorder, but there was no scientific evidence suggesting an identifiable threshold above which the risk existed…” (G/TBT/M/60)  “The representative of the European Union reiterated concerns about Canada's Bill C-32 amending the Tobacco Act… the European Union urged Canada to provide some background with regards to its approach to ban a comprehensive list of additives, including certain flavours which might be perceived as appealing to youngsters. Further, the representative of the European Union asked whether Canada could make available scientific studies or other relevant information that established a link between the prohibited additives and attractiveness to youngsters.” (G/TBT/M/51) |
|  | Other harmful consequences | The measure may have adverse non-trade consequences that may have not been adequately assessed or taking into account, including misleading consumers, a rise in illicit trade, exacerbating the problem, and having a negative impact on small businesses, incomes, employment, and the wider economy | “…the EU was concerned with the introduction by this draft regulation of two different types of warnings on alcoholic consumption…could mislead consumers, who could conclude that some alcoholic beverages were more harmful than others” (G/TBT/M/58)  “The representative of the European Union expressed her delegation's concerns regarding the procedure of notification of alcoholic products... she stated that the labelling requirements were excessive and in some cases could mislead the consumer…” (G/TBT/M/59) |
|  | Policy principle | Members question the principles behind the policy, such as whether it was proportionate with the risks associated with the product(s) | “The representative of Zambia echoed concerns regarding Australia’s measure… Mandatory plain packaging was a disproportionate response to the stated health objectives of the legislation.” (G/TBT/M/55) |
|  | Intellectual property | The policy is inconsistent with TRIPS and/or would potentially require businesses to share confidential information and their intellectual property | “Honduras argued that the measure in question not only lacked basis in scientific proof, but was also more trade restrictive than necessary and damaged intellectual property rights…” (G/TBT/M/62) |
|  | International practice | The policy was inconsistent with existing regulations and practices (but not a formally agreed international standard) in place in other countries | “The representative of the European Union stated that in January 2016 the EU had provided detailed comments on India's draft alcoholic beverages regulation… He outlined several concerns on the notified draft. It was considered that, if adopted in its current form, the draft regulation would create a number of unnecessary barriers to trade, particularly in consideration of its inconsistencies with current international practice. |
