# Supplementary material for: Trade challenges at the World Trade Organization to national noncommunicable disease prevention policies: A thematic document analysis of trade and health policy space
Source: PLoS Med. 2018 Jun 26;15(6):e1002590. doi: 10.1371/journal.pmed.1002590 (PMC6019096; doi:10.1371/journal.pmed.1002590)
Supplement: S1 Table — (DOCX) [file pmed.1002590.s002.docx]

**S1 Table. Summary of trade challenges to food, beverage and tobacco regulations**

| ID | Raised against | Raised by | Date of first meeting | Regulation | Policy stage | Products regulated |
| --- | --- | --- | --- | --- | --- | --- |
| 93 | Bolivia | Canada, Guatemala, United States, European Union | 15/06/11 | Labelling; Marketing | In force | Food products and ingredients |
| 92 | China | Japan, Korea, Rep., New Zealand, United States, European Union | 09/03/16 | Registration | Draft | Infant milk formula |
| 91 | Egypt, Arab Rep. | Australia, Switzerland, Turkey, Ukraine, United States, European Union, South Africa, Chile, Canada, China, Norway | 20/06/02 | Registration | In force | Cosmetics; Food products and ingredients; Non-alcoholic beverages; Furniture and utensils; Electrical machinery and devices |
| 90 | European Union | Indonesia | 07/11/03 | Prohibition | Ratified | Tobacco and tobacco products |
| 89 | Hungary, European Union | Cuba, Dominican Republic, Guatemala, Indonesia, Nigeria | 01/07/08 | Labelling; cap | Proposed | Tobacco and tobacco products |
| 88 | India | Australia, Canada, Chile, Guatemala, Japan, Mexico, New Zealand, South Africa, Switzerland, United States, European Union | 01/07/08 | Labelling; definitions | Draft | Alcoholic beverages |
| 87 | Kenya | South Africa, United States, European Union | 24/03/10 | Standards and restrictions | Draft | Alcoholic beverages |
| 86 | Russian Federation | Ukraine | 03/11/10 | Prohibition | In force | Food products and ingredients; Alcoholic beverages; Non-alcoholic beverages |
| 85 | Russian Federation | Indonesia | 24/03/10 | Excise tax | Ratified | Food products and ingredients; Soft-drinks |
| 84 | South Africa | Canada, Guatemala, European Union | 24/03/11 | Labelling | Proposed | Alcoholic beverages |
| 83 | Thailand | United States, Australia, New Zealand, European Union | 24/03/11 | Marketing | Draft | Infant milk formula |
| 82 | Canada | Indonesia | 05/11/09 | Prohibition | Ratified | Tobacco and tobacco products |
| 81 | European Union | India | 25/06/09 | Standards and restrictions | In force | Food products and ingredients |
| 80 | Indonesia | Australia, Brazil, Canada, European Union | 18/03/15 | Prohibition | In force | Food products and ingredients |
| 79 | Norway | Cuba, Dominican Republic, Indonesia, Zimbabwe | 06/03/13 | Labelling | Draft | Tobacco and tobacco products |
| 78 | Russian Federation | Ukraine | 20/06/02 | Prohibition | In force | Food products and ingredients |
| 77 | Singapore | Dominican Republic, Guatemala, Indonesia | 22/12/05 | Labelling | Proposed | Tobacco and tobacco products |
| 76 | Ecuador | Brazil, Canada, Chile, Costa Rica, United States, European Union | 09/11/07 | Standards and restrictions; CAP | In force | Cosmetics; Food products and ingredients; Soap and surfuctants |
| 75 | Ecuador | Brazil, Canada, Chile, Colombia, Costa Rica, Guatemala, Mexico, Peru, Switzerland, United States, European Union | 10/11/11 | Labelling | Ratified | Food products and ingredients |
| 74 | Ecuador | Panama | 09/03/16 | Standards and restrictions; CAP; Labelling | Draft | Food products and ingredients |
| 73 | Ecuador | Brazil, Canada, United States, European Union | 18/03/09 | Labelling | Draft | Food products and ingredients |
| 72 | Ecuador | Canada, Chile, Mexico, United States, European Union | 23/06/10 | Labelling | Draft | Alcoholic beverages |
| 71 | European Union | Cuba, Dominican Republic, Honduras, Indonesia, Malawi, Nicaragua, Nigeria, Zimbabwe, Ukraine | 24/03/11 | Labelling | Proposed | Tobacco and tobacco products |
| 70 | Mexico | Chile, United States, European Union | 20/03/12 | Labelling | Draft | Alcoholic beverages |
| 69 | Moldova | Ukraine | 30/10/13 | Labelling | Proposed | Tobacco and tobacco products |
| 68 | Russian Federation | Ukraine | 19/03/14 | Prohibition | In force | Alcoholic beverages |
| 67 | Saudi Arabia | Switzerland, United States, European Union | 19/03/14 | Labelling; Marketing | Proposed | Soft-drinks |
| 66 | South Africa | New Zealand, European Union | 19/03/14 | Labelling; Marketing | Proposed | Food products and ingredients |
| 65 | Thailand | Australia, Canada, Chile, Guatemala, Japan, Mexico, New Zealand, South Africa, United States, European Union | 19/03/14 | Labelling | Draft | Alcoholic beverages |
| 64 | European Union | Cuba, Dominican Republic, Guatemala, Honduras, Indonesia, Malawi, Nicaragua, Nigeria, Zimbabwe, Ukraine | 18/06/14 | Labelling | Proposed | Tobacco and tobacco products |
| 63 | Chile | Argentina, Australia, Brazil, Canada, Colombia, Costa Rica, Guatemala, Mexico, Peru, Switzerland, United States, European Union | 15/09/98 | Labelling | Draft | Food products and ingredients |
| 62 | Ecuador | Canada, Mexico, United States, European Union | 15/06/16 | Labelling | Ratified | Alcoholic beverages |
| 61 | European Union | Cuba, Dominican Republic, Guatemala, Honduras, Indonesia, Malawi, Nicaragua, Nigeria, Zimbabwe, Ukraine | 10/11/11 | Labelling | Proposed | Tobacco and tobacco products |
| 60 | European Union | Cuba, Dominican Republic, Guatemala, Honduras, Indonesia, Malawi, Mexico, Mozambique, Nicaragua, Nigeria, Philippines, Zimbabwe, Ukraine, Zambia | 20/06/97 | Labelling; Marketing; Standards and restrictions | Proposed | Tobacco and tobacco products |
| 59 | Indonesia | Australia, Brazil, Canada, Guatemala, Mexico, Switzerland, United States, European Union | 29/06/01 | Labelling | Ratified | Food products and ingredients |
| 58 | Peru | Argentina, Brazil, Canada, Colombia, Costa Rica, Guatemala, Mexico, Switzerland, United States, European Union | 09/11/06 | Labelling; Marketing; Education program | Draft | Food products and ingredients |
| 57 | Turkey | Canada, Mexico, United States, European Union | 05/11/08 | Labelling | In force | Alcoholic beverages |
| 56 | Ukraine | United States | 20/03/08 | Marketing | Ratified | Tobacco and tobacco products; Alcoholic beverages |
| 55 | Dominican Republic | Mexico, European Union | 17/06/13 | Definitions; Labelling | Draft | Alcoholic beverages |
| 54 | Indonesia | Mexico, South Africa | 06/03/13 | Definitions | Draft | Alcoholic beverages |
| 53 | Israel | Argentina, Mexico, United States, European Union | 05/11/14 | Labelling; Marketing | Draft | Alcoholic beverages |
| 52 | New Zealand | Cuba, Dominican Republic, Guatemala, Honduras, Indonesia, Malawi, Mexico, Nicaragua, Nigeria, Zimbabwe, Ukraine, Zambia | 04/11/15 | Labelling | Proposed | Tobacco and tobacco products |
| 51 | Russian Federation | Argentina, Australia, Guatemala, Mexico, New Zealand, South Africa, Ukraine, United States, European Union | 15/06/16 | Definitions; Registration; CAP | Draft | Alcoholic beverages |
| 50 | Vietnam | Australia, Canada, Chile, Mexico, New Zealand, South Africa, United States, European Union | 09/03/16 | Labelling | Draft | Alcoholic beverages |
| 49 | Vietnam | Australia, Canada, Chile, New Zealand, United States, European Union | 15/03/02 | Labelling; CAP | Ratified | Food products and ingredients |
| 48 | Australia | Chile, China, Colombia, Cuba, Dominican Republic, El Salvador, Guatemala, Honduras, Hong Kong SAR, China, China, Indonesia, Japan, Mexico, Nicaragua, Nigeria, Philippines, Russian Federation, Zimbabwe, Switzerland, Turkey, Ukraine, Zambia, European Union | 18/03/09 | Labelling | Draft | Tobacco and tobacco products |
| 47 | Brazil | Chile, Colombia, Cuba, Dominican Republic, Ecuador, Guatemala, Honduras, Indonesia, Jordan, Kenya, Malawi, Mexico, Mozambique, Nicaragua, Nigeria, Philippines, Russian Federation, Zimbabwe, Turkey, The former Yugoslav Republic of Macedonia, Tanzania, Zambia, European Union | 24/03/11 | Standards and restrictions | Draft | Tobacco and tobacco products |
| 46 | Brazil | Norway, Peru, European Union | 09/03/16 | Standards and restrictions; CAP | Draft | Food products and ingredients |
| 45 | China | United States, European Union | 13/06/12 | Labelling | In force | Food products and ingredients |
| 44 | Colombia | United States, European Union | 17/06/13 | Production, distribution and disposal; Marketing; Labelling | Draft | Alcoholic beverages |
| 43 | El Salvador | Mexico | 18/03/15 | Standards and restrictions; Marketing | In force | Non-alcoholic beverages |
| 42 | India | Australia, Canada, Chile, Japan, New Zealand, Switzerland, United States, European Union | 05/07/07 | Labelling | Draft | Food products and ingredients |
| 41 | Kenya | Mexico, United States, European Union | 27/11/12 | Labelling | In force | Alcoholic beverages |
| 40 | Mexico | United States, European Union | 15/06/11 | Labelling | Draft | Non-alcoholic beverages; Soft-drinks |
| 39 | United States | Mexico | 15/06/16 | Standards and restrictions; Registration; CAP | Ratified | Food products and ingredients |
| 38 | Vietnam | Australia, Canada, Chile, New Zealand, United States, European Union | 02/07/03 | Standards and restrictions; CAP | Ratified | Cosmetics; Alcoholic beverages; Electrical machinery and devices |
| 37 | Brazil | Mexico, United States, European Union | 25/06/09 | Standards and restrictions; Labelling | Draft | Alcoholic beverages |
| 36 | Brazil | Switzerland, United States, European Union | 10/11/11 | Labelling; CAP | Ratified | Food products and ingredients |
| 35 | Brazil | Mexico | 05/11/14 | Registration; Marketing | Draft | Food products and ingredients |
| 34 | Colombia | European Union | 09/11/07 | Standards and restrictions | In force | Infant milk formula |
| 33 | Thailand | Argentina, Australia, Canada, Chile, Mexico, New Zealand, Switzerland, United States, European Union | 18/06/14 | Labelling | Proposed | Alcoholic beverages |
| 32 | Vietnam | Australia, Chile, Mexico, United States, European Union | 11/06/99 | Standards and restrictions; Labelling | Draft | Alcoholic beverages |
| 31 | Canada | Argentina, Brazil, Burundi, Chile, Colombia, Croatia, Cuba, Dominican Republic, Ecuador, Guatemala, Honduras, Indonesia, Japan, Jordan, Kenya, Malawi, Mexico, Mozambique, Philippines, Zimbabwe, Switzerland, Turkey, Uganda, The former Yugoslav Republic of Macedonia, Egypt, Arab Rep., Tanzania, United States, Zambia, European Union | 27/11/12 | Prohibition | In force | Tobacco and tobacco products |
| 30 | Canada | European Union | 17/06/15 | Labelling | Draft | Food products and ingredients |
| 29 | Colombia | United States, European Union | 22/12/05 | Standards and restrictions | Draft | Alcoholic beverages |
| 28 | India | United States, European Union | 17/06/13 | Labelling | Draft | Food products and ingredients |
| 27 | Korea, Rep. | China, European Union | 20/03/12 | Labelling | In force | Food products and ingredients |
| 26 | Brazil | United States, European Union | 05/11/14 | CAP | Draft | Alcoholic beverages |
| 25 | Brazil | Mexico, United States, European Union | 17/06/15 | Definitions; Standards and restrictions | Draft | Alcoholic beverages |
| 24 | European Union | Bolivia, Brazil, Colombia, Costa Rica, Cuba, Ecuador, Mexico, Peru, Venezuela, RB | 15/06/16 | CAP | Draft | Food products and ingredients |
| 23 | European Union | Argentina, Cuba, Ecuador | 09/03/16 | Labelling; production, distribution and disposal | Draft | Food products and ingredients |
| 22 | South Africa | United States | 05/11/14 | Labelling; Marketing | Proposed | Food products and ingredients |
| 21 | United States | European Union | 04/11/15 | Standards and restrictions | Proposed | Food products and ingredients |
| 20 | China | European Union | 20/03/08 | Standards and restrictions; Labelling | Draft | Alcoholic beverages |
| 19 | Israel | United States | 05/11/14 | Standards and restrictions; CAP | In force | Infant milk formula |
| 18 | Moldova | European Union | 09/03/16 | Labelling; CAP; Standards and restrictions | Ratified | Non-alcoholic beverages; Soft-drinks |
| 17 | Thailand | Australia, Canada, United States, European Union | 21/03/07 | Labelling | Draft | Food products and ingredients |
| 16 | United States | Argentina | 24/03/10 | Labelling; Marketing | Proposed | Alcoholic beverages |
| 15 | European Union | China | 18/06/14 | CAP | Proposed | Food products and ingredients |
| 14 | Uruguay | European Union | 09/03/16 | Standards and restrictions | In force | Food products and ingredients |
| 13 | China | United States | 30/10/13 | Labelling; CAP | Proposed | Food products and ingredients |
| 12 | Peru | United States | 06/03/13 | Marketing; production, distribution and disposal | Ratified | Infant milk formula |
| 11 | Brazil | Barbados, Dominican Republic, Jamaica, Trinidad and Tobago, United States, European Union | 19/03/14 | Labelling; definitions; CAP; Standards and restrictions | In force | Alcoholic beverages |
| 10 | Korea, Rep. | Iceland, New Zealand, Norway, European Union | 28/06/96 | Prohibition | In force | Food products and ingredients |
| 9 | Brazil | European Union | 21/06/02 | Labelling; CAP | In force | Alcoholic beverages |
| 8 | China | Korea, Rep., European Union | 09/11/07 | Labelling; CAP | In force | Food products and ingredients; Cosmetics |
| 7 | India | European Union | 05/11/08 | Standards and restrictions | Proposed | Food products and ingredients |
| 6 | United States | Argentina, Australia, Brazil, Canada, China, Mexico, New Zealand | 24/03/11 | Labelling | Draft | Food products and ingredients |
| 5 | European Union | Argentina, Australia, Brazil, Canada, United States | 09/11/06 | Labelling; CAP | Proposed | Food products and ingredients |
| 4 | New Zealand | Canada | 23/06/10 | Labelling; CAP | Proposed | Food products and ingredients |
| 3 | Egypt, Arab Rep. | United States, European Union | 15/06/11 | Labelling | In force | Food products and ingredients |
| 2 | European Union | Canada, United States | 13/06/12 | Labelling | In force | Food products and ingredients |
| 1 | United States | European Union | 13/06/12 | Standards and restrictions | Proposed | Non-alcoholic beverages |

*Notes*: See tables S1, S3 and S4 or description of category codes.
