# Supplementary material for: Trade challenges at the World Trade Organization to national noncommunicable disease prevention policies: A thematic document analysis of trade and health policy space
Source: PLoS Med. 2018 Jun 26;15(6):e1002590. doi: 10.1371/journal.pmed.1002590 (PMC6019096; doi:10.1371/journal.pmed.1002590)
Supplement: S2 Table — (DOCX) [file pmed.1002590.s003.docx]

**S2 Table. Measures introduced by countries to protect health that were subject to STCs**

| Measure | Description |
| --- | --- |
| Conformity assessment procedures | The methods used to evaluate, test and certify whether a product meets the country’s standards |
| Standards | Product standards, including quality requirements, and limits to the use of certain materials |
| Labelling | Packaging and labelling requirements and restrictions |
| Prohibition | Product ban applying both to importation and domestic production |
| Registration | Regulations for registering a product for sale or production in a country |
| Marketing | Rules and restrictions on advertising and promotion of the product |
| Definitions | Product definitions |
| Production, distribution and disposal | Regulations about production processes, transportation of goods, and disposal of waste |
| Education | Health promoting education campaign |

*Notes:* Policies were coded by first extracting the full description of the policy from the WTO TBT-IMS database and TBT Committee Minutes, writing a short summary of the measure, and then aggregating policies into common categories.
