# Supplementary material for: Trade challenges at the World Trade Organization to national noncommunicable disease prevention policies: A thematic document analysis of trade and health policy space
Source: PLoS Med. 2018 Jun 26;15(6):e1002590. doi: 10.1371/journal.pmed.1002590 (PMC6019096; doi:10.1371/journal.pmed.1002590)
Supplement: S3 Table — (DOCX) [file pmed.1002590.s004.docx]

**S3 Table. Issues raised in informal challenges about measures introduced to protect health**

| Issue | Description |
| --- | --- |
| Discrimination | Concern about measure seen as discriminatory against foreign producers |
| Information | Request for further information and clarification about the content and scope of the measure |
| Standards | Concern about consistency of measure with international standards (WTO rules state that measures should conform with international standards unless members can make a case that the standard should not apply; TBTs are immune from formal disputes if the measure conforms to a standard) |
| Time | Concern about the amount of time given to adapt to the measure and the extent to which this length of time constitutes a 'reasonable interval' |
| Transparency | Concern about lack of notification of a measure and details of its implementation, lack of an enquiry point, inadequate access to the final text of the regulation, or inadequate time to review and adapt to the new regulation. |
| Rationale | Concern about rationale for introducing the measure and whether it was legitimate |
| Unnecessary barrier to trade | Concern that the measure is unnecessarily trade restrictive i.e. an alternative measure could be introduced that achieves the stated objective but is less trade restrictive |
| Technical assistance | Concern about a developing country’s need for technical assistance in developing standardising bodies, assessment of conformity with standards, and the establishment and functioning of institutions and the legal framework to conform to WTO obligations |
| Special treatment | Concern that the measure does not take into account the special development, financial and trade needs of developing countries |
| Non-product related production and process method (NPR PPM) | Concern that the measure regulates a process or production method in which the method itself leaves no trace in the final product |
| Other issues | Concern about some aspect of the measure that is not a specific violation of the TBT measure, such as the adequacy of its scientific basis, and whether it was proportionate with the risks posed by a product (see Appendix 1 for full details) |

*Notes:* Issues are selected from pre-defined categories by the countries who raise the informal WTO challenges. Each issue category reflects a way in which a measure can be deemed incompatible with WTO rules according to the TBT Agreement. However, ‘other issues’ do not refer to a violation of the TBT agreement. We manually coded the content of these issues (see Appendix 1 for full details).
